# Simulation‐Based Mastery Learning Improves Emergency Medicine Residents' Ability to Perform Emergency Cricothyrotomy

**DOI:** 10.1002/aet2.70124

**Published:** 2026-01-28

**Authors:** Dana E. Loke, Andrew M. Rogers, David H. Salzman

**Affiliations:** ^1^ BerbeeWalsh Department of Emergency Medicine University of Wisconsin School of Medicine and Public Health, 800 University Bay Drive Madison Wisconsin USA; ^2^ Department of Emergency Medicine, Endeavor Health Evanston Illinois USA; ^3^ Department of Emergency Medicine and Department of Medical Education Northwestern University, Feinberg School of Medicine Chicago Illinois USA

**Keywords:** checklist, competency‐based education, emergency cricothyrotomy, mastery learning, simulation

## Abstract

**Background:**

Emergency cricothyrotomy (EC) is a critical procedure for Emergency Medicine (EM) physicians to master. Simulation‐based mastery learning (SBML), a form of competency‐based education with deliberate practice, has been shown to prepare residents to perform numerous procedures. The objectives of this study were to create a SBML curriculum to teach EM residents EC, to compare pre‐ and post‐training scores for EM residents performing EC, and to assess retention of mastery at 5 months.

**Methods:**

EM residents completed baseline testing, training, post‐testing, and retention testing using a commercially available task trainer and completed a post‐curriculum satisfaction survey. An expert panel of EM physicians and trauma surgeons reviewed a previously developed 27‐item checklist and set a minimum passing standard (MPS) using a Mastery Angoff approach. “Mastery” was defined as a checklist score meeting or exceeding the MPS.

**Results:**

The MPS was set at 26 correctly performed items (96.3%). A cohort of 56 EM residents completed the curriculum. No resident achieved mastery on baseline testing; 33 residents (59.0%) achieved mastery on initial post‐testing. Checklist scores significantly improved from baseline to post‐testing. All residents that did not initially achieve mastery successfully did after an additional 30‐Min of deliberate practice. At 5 months, 40 residents retained mastery (71.4%). Retention performance of various checklist items decayed more than others. Retention performance did not significantly vary based on PGY level or if additional practice was required to achieve mastery. Post‐curriculum survey data showed high resident satisfaction and significantly higher confidence in performing emergency cricothyrotomy.

**Conclusion:**

A SBML curriculum improved EM residents' ability to perform EC in a simulated environment. Baseline testing highlighted the gap that traditional training approaches have in teaching this critical skill to a mastery standard. Retention results can inform a timeline for additional deliberate practice to ensure continual mastery.

## 1 Introduction

Emergency cricothyrotomy (EC) is a critically important procedure in which Emergency Medicine (EM) physicians must demonstrate competence [1]. There are few opportunities to learn EC in the clinical environment and experienced EM physicians feel inadequately trained [2, 3, 4]. On‐shift learning is complicated due to potential consequences for patient safety. Historically, the study site program's method for teaching EC involved a non‐standardized annual “rare procedures” simulation session, not following a competency‐based training model.

**FIGURE 1:**
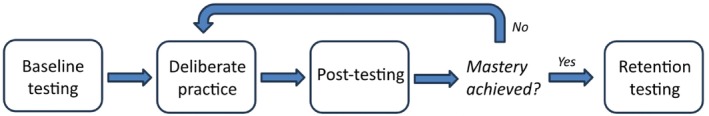
Mastery learning paradigm.

We hypothesized that an educational curriculum designed to ensure competency using simulation‐based mastery learning (SBML) would better meet this training need for EC. SBML is a rigorous form of competency‐based education to ensure demonstration of a skill to a mastery level (Figure 1) [5]. SBML has a consistent record of improving resident performance on procedural skills and has been demonstrated to improve skill acquisition, patient outcomes, and healthcare costs across EM‐relevant skills [6, 7, 8, 9, 10, 11, 12, 13, 14, 15, 16, 17].

The objectives of this study were to create a curriculum using SBML literature‐based approach to teach EM residents EC, to compare pre‐ and post‐training scores for EM residents performing EC on a task trainer, and to assess retention of EC procedure mastery.

## 2 Methods

### 2.1 Study Setting & Population

The study was performed at a single urban academic center with a 4‐year EM residency training program. Four residents were excluded from the study due to participation in the checklist design and assessment process. The curriculum was part of the required annual residency simulation curriculum; however, participation in the study was optional. Informed written consent was obtained. Assessments occurred in the simulation center using a procedural task trainer (TraumaMan, Simlab, Seattle, WA) from 8/31/21 to 6/21/22. The study was determined to be exempt by the IRB at Northwestern University, Feinberg School of Medicine.

### 2.2 Standard Setting

This study used a previously developed checklist following guidance that was subsequently published [18]. The checklist demonstrated strong inter‐rater reliability [10]. A MPS was determined using an expert panel who did not participate in checklist creation. The panel included EM and Trauma Surgery physicians of varying practice type and location (7 academic, 5 community/5 internal study site, 7 external), and gender (4 female, 8 male). Experts were blinded to each other's identities and responses. The panel set a MPS using a Mastery Angoff approach. Scores were averaged and a MPS was calculated. Mastery was defined as the participant achieving a score≥MPS.

### 2.3 Baseline Assessment

The curriculum included all essential components of the mastery learning paradigm (Figure 1). Participants completed pre‐ and post‐curriculum surveys, rating confidence in performing EC (scale of 0 (no confidence) to 100 (extremely confident)). Surveys and checklists were administered using Qualtrics (Qualtrics, Seattle, WA).

Participants completed baseline testing and were assessed by an in‐person rater blinded to the MPS using the 27‐item checklist. The checklist involved a standardized prompt, orienting the resident to a scenario of a patient that could not be intubated, oxygenated, or ventilated and required an EC.

### 2.4 Curriculum Intervention

Residents not meeting mastery at baseline testing watched a 12‐min procedural video produced by the study team and attended a 30‐min individual in‐person session to review baseline performance and practice the procedure with a trained facilitator who was also the primary rater. Residents were not given access to the checklist at any time.

### 2.5 Post‐Testing

About 10 weeks after deliberate practice, participants completed initial post‐testing. Participants not meeting or exceeding the MPS had additional opportunities for deliberate practice and post‐testing until mastery.

### 2.6 Retention Testing

Participants underwent retention testing 5‐months after post‐testing to assess skill decay and completed a survey to gauge satisfaction.

### 2.7 Statistical Analysis

Baseline data were previously analyzed [10]. Differences in scores between baseline and initial post‐testing and between baseline and retention testing were determined using the Wilcoxon signed‐rank test (*z*‐score). Analysis of variance was used to compare retention performance between PGY cohorts. Chi‐square test was used to compare retention based on need for additional practice to achieve mastery. Logistic regression was used to compare pre‐test and final post‐test scores with likelihood of retention.

Mean Likert scores with standard deviations were calculated for the post‐curriculum survey. Pre‐ and post‐curriculum resident confidence was analyzed using Wilcoxon signed‐rank test. All data were analyzed using Microsoft Excel 16.97.2 (Microsoft Corporation, Redmond, WA).

## 3 Results

**FIGURE 2:**
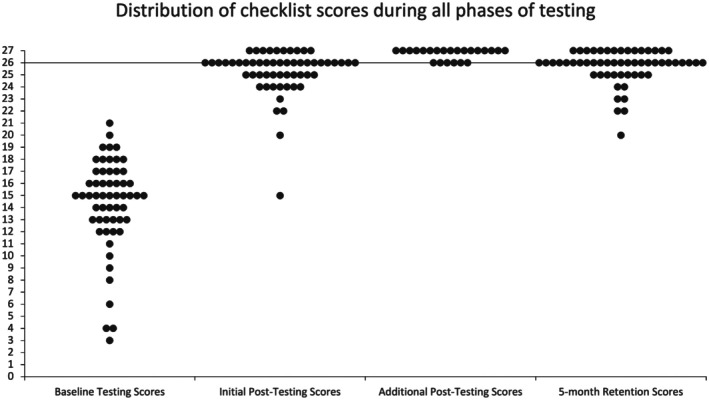
Distribution of checklist scores during each phase of testing.

All 56 eligible residents consented and completed the study, including 15 PGY‐1 s, 14 PGY‐2 s, 13 PGY‐3 s, and 14 PGY‐4 s. The expert panel set the MPS at 26/27 correctly performed items (96.3%). Table 1 highlights learner performance for individual checklist items across phases of the study, including baseline data published previously [10]. No resident achieved mastery on baseline testing (Figure 2). The mean score at baseline was 14.3 (95% CI 13.3–15.3).

**TABLE 1:** Successful learner performance for individual checklist items.

Checklist item		Baseline testing (*n* = 56)^a^		Initial Post‐testing (*n* = 56)^a^		Additional post‐testing (*n* = 23)^a^		Retention testing (*n* = 56)^a^	
		*n* ^b^	(%)^b^	*n* ^b^	(%)^b^	*n* ^b^	(%)^b^	*n* ^b^	(%)^b^
1.	Gathers sterile supplies	27	48.2	54	96.4	23	100	55	98.2
2.	Gathers primary cricothyrotomy procedure supplies	37	66.1	56	100	22	95.7	55	98.2
3.	Gathers secondary/supplemental cricothyrotomy procedure supplies	46	82.1	56	100	23	100	56	100
4.	Gathers supplemental intubation supplies	0	0	27	48.2	22	95.7	31	55.4
5.	Washes hands	10	17.9	43	76.8	23	100	55	98.2
6.	Sterilizes the neck	49	87.5	54	96.4	22	95.7	56	100
7.	Dons PPE	38	67.9	56	100	23	100	55	98.2
8.	Proceduralist positions on the patient's right side	50	89.3	56	100	23	100	56	100
9.	Identifies cricothyroid membrane (CTM)	27	48.2	55	98.2	22	95.7	56	100
10.	Uses thumb and middle finger of non‐dominant hand to stabilize airway	19	33.9	50	89.3	22	95.7	51	91.1
11.	Confirms incision site with palpation by index finger on the CTM using non‐dominant hand while maintaining stabilization using thumb and middle finger of non‐dominant hand	16	28.6	51	91.1	23	100	49	87.5
12.	Uses scalpel to make vertical skin incision ~2–4 cm in length over the CTM using dominant hand	32	57.1	54	96.4	23	100	55	98.2
13.	Dissects down to CTM	49	87.5	54	96.4	23	100	56	100
14.	Re‐identifies CTM by palpation or visualization	43	76.8	55	98.2	23	100	55	98.2
15.	Makes ~1–2 cm (width of scalpel blade) horizontal incision through CTM with dominant hand and maintains scalpel blade in trachea	29	51.8	53	94.6	23	100	54	96.4
16.	Maintains patency of tract	7	12.5	54	96.4	23	100	54	96.4
17.	Removes scalpel, only after tracheal hook, Trousseau dilator, bougie, or secondary scalpel handle is in place, maintaining patency of CTM	7	12.5	54	96.4	23	100	54	96.4
18.	Proceduralist dilates CTM	2	3.6	51	91.1	22	95.7	49	87.5
19.	Inserts ETT or trach	51	91.1	56	100	23	100	56	100
20.	Inserts ETT or trach to correct depth	12	21.4	51	91.1	23	100	54	96.4
21.	Inflates the cuff with a 10 cc syringe	44	78.6	55	98.2	23	100	56	100
22.	Connects BVM to ETT/trach and begins assisted ventilation	52	92.9	56	100	23	100	56	100
23.	Uses capnography to confirm tube location	50	89.3	56	100	23	100	56	100
24.	Listens for bilateral breath sounds	37	66.1	51	91.1	23	100	51	91.1
25.	Secures ETT/trach	36	64.3	54	96.4	23	100	48	85.7
26.	Orders chest X‐ray	26	46.4	55	98.2	23	100	56	100
27.	Documents procedure	5	8.9	46	82.1	23	100	53	94.6

^a^ The total number of participants during each phase of the curriculum.

^b^ The number (*n*) and percentage (%) of learners who performed each checklist item correctly during each phase of testing.

After initial post‐testing, 33 residents (59.0%) achieved mastery. Checklist scores significantly improved from baseline (mean 14.3, 95% CI 13.3–15.3) to initial post‐testing (mean 25.2, 95% CI 24.7–25.8) (*p* < 0.00001). The mean initial post‐testing score for those that did not achieve mastery on initial post‐testing was 23.7 (95% CI 22.7–24.7), compared to 26.3 (95% CI 26.1–26.5) for those that did. Those that did not achieve mastery on initial post‐testing (*n* = 23) were successful after an additional 30 min of deliberate practice, with a mean checklist score of 26.7 (95% CI 26.5–26.9). Checklist items commonly performed incorrectly on initial post‐testing included items 4, 5, 10, and 27 which were all performed less than 90% of the time.

At retention testing, 71.5% (40 of the 56 of residents) retained mastery (Figure 2). Checklist scores were still significantly improved from baseline (mean 26.2, 95% CI 25.1–27.4) (*p* < 0.00001). Retention performance did not significantly vary between PGY cohorts (*p* = 0.72). There was no significant difference in retention between groups requiring additional practice to achieve mastery (*p* = 0.10). Pre‐ and initial post‐test scores did not significantly predict retention (*p* = 0.205 and *p* = 0.624, respectively). Checklist items that were most commonly performed incorrectly during retention testing included items 4, 11, 18, and 25, which were all performed less than 90% of the time.

The post‐curriculum survey indicated high levels of satisfaction with the curriculum. 95% strongly agreed that the curriculum was a beneficial addition to the residency curriculum (mean 4.96, SD 0.19) and 91% strongly desired to include it in the simulation curriculum (mean 4.91, SD 0.29). 95% strongly agreed that the curriculum would positively impact their clinical practice (mean 4.95, SD 0.23). Resident confidence in performing EC improved (22.9 vs. 80.71, *p* < 0.00001).

## 4 Discussion

This SBML curriculum improved EM residents' ability to perform EC on a task trainer. The inability of any resident to meet the MPS during baseline testing highlights gaps in traditional teaching to reach mastery of this critical skill. Perhaps the rigorous MPS established by the expert panel resulted in the inability of any resident to pass the baseline assessment. However, the high MPS is consistent with other studies that developed SBML curricula for EM procedures [8, 9, 10]. All residents ultimately met the mastery standard, which is an expectation of a SBML curriculum. These findings and number of participants requiring additional practice are consistent with previous studies [8, 9, 10].

Retention results showed skill decay at 5 months, which is similar to prior studies [19, 20, 21, 22]. In our study, only one checklist item (item 4) was consistently incorrectly performed in both initial post‐testing and retention testing. This may relate to the complexity of this item compared to others. The other three commonly incorrectly performed items differed from initial post‐testing to retention testing. This may relate to resident self‐reflection and self‐guided learning after post‐testing.

The skill decay noted highlights challenges in maintenance of skills in training and independent practice. Many factors may impact the decay of specific skills including the frequency at which they are practiced or performed in the clinical environment. In this cohort, there was no association of retention with PGY level, pre‐test or initial post‐test score, or whether additional deliberate practice was needed. Additional investigation is needed to consider the rate at which skills decay to ensure that EM physicians maintain mastery of clinical skills, and that timely skill refreshers are provided when needed. The skill decay rate for this procedure has important implications during training and beyond, including the shift to competency‐based assessment within the upcoming ABEM oral boards examination changes.

Despite the increased amount of time compared to traditional approaches, learner satisfaction was exceptionally high. Participants recognized the value this educational approach had for learning this critical procedure.

This study has limitations. It was performed at a single site with a relatively small number of learners, requiring significantly more faculty time than the prior training approach. The task‐trainers did not simulate a bloody field that may be an obstacle in the clinical environment. This SBML curriculum included EM residents only and generalizability for learners should be further explored. We only evaluated retention at 5 months due to academic year constraints and evaluation at additional times may help inform when additional skill refreshers are necessary.

## 5 Conclusion

We developed a SBML curriculum that improved EM residents' ability to perform EC in a simulated environment. Learner satisfaction was exceptionally high. Retention results showed some skill decay at 5 months highlighting the need for ongoing study to ensure maintenance of procedural skill competency.

## Author Contributions

D.E.L. contributed to study concept and design, acquisition of the data, analysis and interpretation of the data, drafting of the manuscript, critical revision of the manuscript. A.M.R. contributed to study concept and design, acquisition of the data, and critical revision of the manuscript. D.H.S. contributed to study concept and design, acquisition of the data, analysis and interpretation of the data, and critical revision of the manuscript.

## Funding

The authors have nothing to report.

## Conflicts of Interest

The authors declare no conflicts of interest.

## Data Availability

The data that support the findings of this study are available from the corresponding author upon reasonable request.
